# Targeting NR4As, a new strategy to fine-tune CAR-T cells against solid tumors

**DOI:** 10.1038/s41392-019-0041-1

**Published:** 2019-03-29

**Authors:** Feng Li, Yi Zhang

**Affiliations:** Biotherapy Center and Cancer Center, The First Affiliated Hospital of Zhengzhou University, Zhengzhou, Henan China

**Keywords:** Cancer therapy, Immunotherapy

In a recent report in *Nature*, Chen et al.^1^ identified the critical transcription factors that drive T-cell dysfunction. Knockout of these transcription factors to augment T-cell activity represents a new approach to enhancing the effectiveness of chimeric antigen receptor T (CAR-T) cell therapy for solid tumors.

CAR-T cells are designer cytotoxic lymphocytes with recognition specificity and tumoricidal potency. Although CAR-T cells exhibit unprecedented effectiveness in treating hematologic malignancies, these cells have limited efficacy against solid tumors.^2^ Therefore, intensive efforts have been made to augment the efficiency of CAR-T cell therapy for solid tumors.

A major barrier in solid tumors is the hostile microenvironment surrounding CAR-T cells. To overcome the immunosuppressive effects within the tumor niche, several tactics have been undertaken. The administration of programmed cell death protein 1 (PD-1) antibodies is effective in restoring CAR-T cell function.^3^ Recently, CAR-T cells armed with a secreted minor fragment derived from the variable domains of a PD-1-blocking antibody were tested.^4^ This design seemed more efficient to augment the functionality of host and nearby T cells than did the full-length antagonist. Soluble transforming growth factor-β (TGF-β) is also important for inhibiting T-cell activation. By introducing signal-switch receptors comprising the TGF-β-binding ectodomain and the T-cell activation endodomain, CAR-T cells are more potently activated in the tumor microenvironment.^5^ The suppressive immune cells represent another obstacle for CAR-T cell therapy. With enforced expression of interleukin (IL)-18, CAR-T cells are more active against tumors by decreasing the populations of suppressive regulatory T (T_reg_) cells and M2 macrophages.^6^

T-cell differentiation is also the determinant for therapeutic efficacy. Less differentiated T cells have better persistence in vivo. Nevertheless, a large portion of T cells are terminally differentiated during CAR-T cell production. To augment the populations of more proliferative T cells with stem-cell-like or memory phenotypes, specific supplements were added in culture. The addition of IL-7 and IL-15 but not IL-2 induces the generation of more memory cells without compromising the cytotoxic function.^7^ Similarly, supplementation with a phosphoinositide 3-kinase-δ (PI3k-δ) antagonist shows promise in preserving memory subsets during CAR-T cell expansion.^8^ Alternatively, an infusion with naive T cells, which subsequently differentiate in vivo, may result in better clinical responses. The combination of CAR-modified induced pluripotent stem cells (iPSCs) and organoid-dictated T-cell lineage commitment makes this hypothesis possible. A previous group established such an approach to produce naive CAR-T cells,^9^ which may introduce new scenarios in treating tumors.

Recently, a report published in *Nature* proposed a new strategy to improve CAR-T cell therapy.^1^ To avoid autoimmunity, various transcription factors are upregulated to limit the intensity and interval of T-cell activation. Such factors will upregulate the expression of inhibitory receptors, subsequently induce terminal differentiation and downregulate the secretion of inflammatory cytokines. Hence, finding the key intrinsic initiator of T-cell dysfunction can provide new targets for the preparation of upgraded CAR-T cells. Chen et al.^1^ uncovered NR4A1, NR4A2 and NR4A3 as the central transcriptional factors that drive T-cell dysfunction. To identify the intrinsic triggers of exhaustion, these authors checked the transcriptional adaptations between functional and hypofunctional CD8^+^ T cells. By analyzing single cell transcriptome and chromatin accessibility, the researchers discovered that the transcription factors NR4A1, NR4A2 and NR4A3 were upregulated and played critical roles in mediating CD8^+^ T-cell exhaustion in mouse CAR-modified and endogenous lymphocytes and their human counterparts exposed to tumor or chronic virus infection. When all three NR4As were deficient, the activated CD8^+^ T cells demonstrated features resembling effector T cells with increased functionality rather than those with an exhausted status. Additionally, the researchers generated CAR-T cells with a deletion of NR4As. Compared with their wild-type counterparts, the NR4A-deficient CAR-T cells showed enhanced cytotoxicity but decreased inhibitory receptor expression and promoted tumor regression more significantly.

**Fig. 1:**
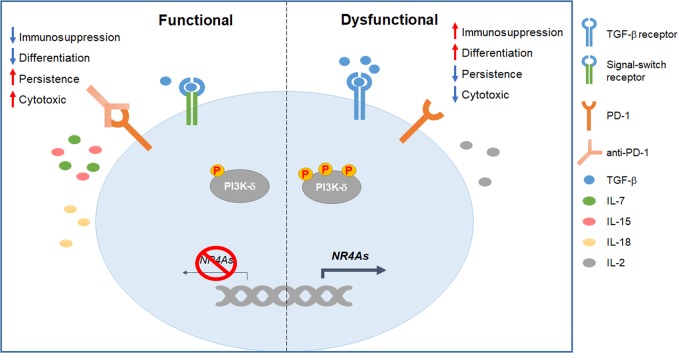
Strategies to augment chimeric antigen receptor T (CAR-T) cell activity in treating solid tumors. Dysfunction of CAR-T cells is related to many intrinsic and extrinsic factors (right). Maintenance of the proliferative T cells and reversion of the inhibition signals are reasonably needed to sustain CAR-T cell function within the tumor (left). Manipulation of the genes that exacerbate T-cell exhaustion is also effective in improving the efficacy of CAR-T cell therapy (left)

This finding provides us with new candidate targets to augment the cytotoxic capacities of CAR-T cells during chronic antigen engagement, which supplements the approaches that help to improve the effectiveness of adoptive transfer therapy for solid tumors (Fig. 1).
